# A Study on Urinary Amylase and Serum Amylase in Diagnosing Acute Pancreatitis

**DOI:** 10.7759/cureus.70809

**Published:** 2024-10-04

**Authors:** Smitha Mogekar, Sudhir Jayakar, Kondapalli Sri Sai Teja Sampath, Vinay Badangi

**Affiliations:** 1 General Surgery, Dr. D. Y. Patil Medical College, Hospital and Research Centre, Dr. D. Y. Patil Vidyapeeth (Deemed to be University), Pune, IND

**Keywords:** acute pancreatitis, diagnosis, pancreatic disease, pancreatic enzymes, pancreatitis management, sensitivity, serum amylase, serum lipase, urinary amylase

## Abstract

Background: Acute pancreatitis is a prevalent condition often characterized by abdominal pain, yet its early diagnosis remains challenging due to the limitations of current diagnostic tests, particularly serum amylase. This study aimed to evaluate the effectiveness of urinary amylase compared to serum amylase in diagnosing acute pancreatitis, with a focus on its sensitivity and prolonged detection capabilities.

Materials and methods: Conducted over two years at a tertiary care hospital, Dr. D. Y. Patil Medical College, Hospital and Research Centre in Pune, India, the study included 60 patients suspected of acute pancreatitis based on clinical and radiological criteria. Participants aged 20 to 60 were included, while those with chronic pancreatitis, pancreatic malignancies, or significant comorbidities were excluded. Serum and urinary amylase levels were measured, and statistical analysis was performed using MS Excel and IBM SPSS version 27 (IBM Corp., Armonk, NY).

Results: The study population had a mean age of 37.90 years, predominantly that is 50 (83.3%) were male. The median serum amylase level was 311 U/L, while urinary amylase levels averaged 501 U/L. Significant findings included urinary amylase levels being significantly higher in females compared to males. Diagnostic imaging revealed various pancreatic conditions, including acute pancreatitis with complications.

Conclusion: Urinary amylase proves to be a valuable diagnostic marker for acute pancreatitis, offering several advantages over traditional serum amylase measurements. Integrating urinary amylase measurement into clinical practice can enhance diagnostic accuracy and provide a more comprehensive approach to diagnosing acute pancreatitis.

## Introduction

Acute pancreatitis is a common condition that is frequently accompanied by acute pain in the abdomen. However, early diagnosis continues to be challenging due to the difficulty in performing rapid tests for pancreatic enzymes in many clinics. The 2015 Japanese guidelines for the management of acute pancreatitis (JPN Guideline 2015) advised the measurement of serum lipase rather than serum amylase for the diagnosis, as serum lipase offers greater specificity for pancreatic enzymes [1]. Traditionally, serum amylase has been used in diagnostic procedures; however, it has a relatively short half-life (approximately 10 to 12 hours) and returns to normal levels within three to five days [2]. This short window can make diagnosing acute pancreatitis difficult, particularly in patients with mild disease or those presenting late. Furthermore, amylase is excreted in the urine, which may continue to be elevated for several days even after serum levels normalize [3].

Given these limitations, urinary amylase measurement might be a more reliable and sensitive diagnostic tool. The clearance of pancreatic enzymes into the urine increases during pancreatitis, making urinary levels potentially more indicative of the condition than serum levels. Additionally, levels of urinary amylase often remain elevated for a longer period compared to serum levels [4].

This study aims to evaluate the effectiveness of urinary amylase levels in diagnosing acute pancreatitis compared to serum amylase. This non-invasive method could offer increased sensitivity and prolonged detection, providing a valuable tool in diagnosing acute pancreatitis. The investigation will compare the sensitivity of serum amylase and urinary amylase and explore the relationship between these two biomarkers.

## Materials and methods

A prospective study was conducted for two years from August 2022 to July 2024 at the Department of General Surgery in a tertiary care hospital, Dr. D. Y. Patil Medical College, Hospital and Research Centre in Pune, India. The study included 60 patients who were clinically suspected of having acute pancreatitis based on presenting symptoms and radiological findings. Patients aged 20 to 60 years of either sex with radiological findings suggestive of acute pancreatitis were included in the study. Patients with chronic pancreatitis, pancreatic disorders (pancreatic malignancy, insulinoma), or salivary gland disorders, like mumps, parotitis, and ductal stenosis, were excluded. Also, patients on anticoagulation or immunosuppressing medication or critically ill and moribund were excluded from the study.

Ethical clearance was obtained from the Institutional Ethics Committee of Dr. D. Y. Patil Vidyapeeth Pune before the commencement of the study. Written and informed consent was obtained from all participants before their inclusion in the study.

Data collection

Data including history and physical examination findings were recorded on a designed proforma. Levels of serum amylase, serum lipase, and urinary amylase were measured and recorded. Serum amylase and urinary amylase were determined using the Kit method with CNP-G3 (2-chloro-4-nitrophenyl-α-maltotrioside) reagent, and serum lipase was measured using the enzyme colorimetric method. The reference values for diagnosis were 28-100 U/L for serum amylase, <60 U/L for serum lipase, and <110 U/L for urinary amylase. Ultrasonography was performed for all patients, and CT scans were done for the patients where the pancreas was not visualized on USG or complications like peri-pancreatic fluid collection (PFC) or pancreatic necrosis was suspected.

Statistical analysis was conducted using MS Excel (Microsoft 365) and IBM SPSS Statistics for Windows, Version 27.0 (Released 2020. IBM Corp., Armonk, NY). Quantitative data was presented using mean and standard deviation for non-skewed data or median and interquartile range (IQR) for skewed data. Categorical data was presented using frequency (percentage). The normality assumption was checked using the Shapiro-Wilk Normality test. Gender groups were compared using two samples t-tests for normally distributed or Mann-Whiteney U test for non-normal data. For all the tests, a p-value of <0.05 (two-tailed) was considered statistically significant.

## Results

The study population consisted of 60 patients, with a mean age of 37.90 ± 10.55 years. The majority of the participants that is 50 (83.3%) were male demonstrating a significant gender disparity in the sample. The occupational distribution of the patients was diverse, reflecting various socioeconomic backgrounds. The largest occupational group was daily laborers, comprising 23 (38.3%) patients. The clinical presentation of the patients varied, with the most common complaints being abdominal pain and vomiting, reported by 27 (45.0%) patients. Regarding co-morbidities, the majority of the patients that are 50 (83.3%) did not have any co-morbid conditions. However, five (8.3%) patients had diabetes mellitus (DM), and another five (8.3%) patients had hypertension (HTN). This suggests the presence of chronic co-morbidities was relatively low in this cohort. Addiction patterns among the patients showed a high prevalence of alcohol use, with 36 (60.0%) patients reporting alcohol consumption. Additionally, seven (11.7%) patients reported both alcohol and smoking addictions. These findings highlight that a significant proportion of the patients had lifestyle factors that could potentially impact their health (Table 1).

**Table 1:** Demographic and clinical details Values represented by ‡ are mean ± SD for quantitative variables and frequency (percentage) for categorical data.

Demographic and clinical details	Descriptive statistic
Age (in years)‡	37.90 ± 10.55
Gender	
Female	10 (16.7%)
Male	50 (83.3%)
Occupation	
Agriculture	5 (8.3%)
Daily laborer	23 (38.3%)
Driver	5 (8.3%)
Housewife	1 (1.7%)
Industrial laborer	5 (8.3%)
Private job	5 (8.3%)
Shop keeper	5 (8.3%)
Student	5 (8.3%)
Teacher	6 (10.0%)
Complaints	
Pain in the abdomen and distension	6 (10.0%)
Pain in the abdomen and vomiting	27 (45.0%)
Pain in the abdomen with vomiting and fever	5 (8.3%)
Pain in the abdomen, bloating and vomiting	6 (10.0%)
Pain in the peri umbilical region with fever	5 (8.3%)
Pain in the umbilicus and vomiting	5 (8.3%)
Pain in the upper abdomen	6 (10.0%)
Co-morbidities	
Diabetes mellitus	5 (8.3%)
Hypertension	5 (8.3%)
None	50 (83.3%)
Addictions	
Alcohol	36 (60.0%)
Alcohol and smoking	7 (11.7%)
None	17 (28.3%)

The biochemical parameters of the study population were assessed to understand the underlying physiological conditions better. The median total leukocyte count (TLC) was 9,400 cells per microliter (µL), with an IQR from 7,675 to 13,400 µL. This range indicates variability in the immune response among patients, reflecting a potential underlying inflammatory or infectious process in some cases. The median total bilirubin level was 0.82 mg/dL, with an IQR of 0.51 to 1.34 mg/dL. These values fall within the normal range, suggesting that most patients did not have significant hepatic dysfunction or biliary obstruction. However, the upper end of the range indicates that a subset of patients had elevated bilirubin levels, which could be indicative of mild hepatic impairment or hemolysis.

The serum amylase levels showed considerable variability, with a median of 311 U/L and an IQR ranging from 94 to 689 U/L. The elevated median value and wide range suggest that many patients had increased serum amylase levels, which is consistent with the clinical presentation of abdominal pain and could be indicative of pancreatitis or other pancreatic disorders. Serum lipase levels were markedly elevated, with a median of 498.5 U/L and an IQR extending from 214.5 to 2,062 U/L. The high median value and broad range of serum lipase levels point to a significant number of patients experiencing acute pancreatitis, as elevated lipase is a key diagnostic marker for this condition. The median urinary amylase level was 501 U/L, with an IQR of 100.5 to 1,327.25 U/L. The elevated urinary amylase levels in many patients further support the diagnosis of pancreatic involvement, as urinary amylase is another marker for pancreatic enzyme secretion and pancreatitis (Table 2).

**Table 2:** Biochemical parameters Values represented are median (Interquartile range) for quantitative variables.

Biochemical parameters	Median interquartile range	Range
TLC (µL)	9,400	7,675 - 13,400
Total bilirubin (mg/dL)	0.82	0.51 – 1.34
Serum amylase (U/L)	311	94 - 689
Serum lipase (U/L)	498.5	214.5 - 2062
Urinary amylase (U/L)	501	100.5 – 1,327.25

The diagnostic imaging results, including ultrasonography and contrast-enhanced computed tomography (CECT) of the abdomen, provide critical insights into the structural abnormalities and pathological changes in the pancreas among the study population. Ultrasonography was performed on all patients, revealing a variety of pancreatic conditions. Bulky pancreas was observed in 14 (23.3%) patients whereas the pancreas was not visualized in five (8.3%). CECT of the abdomen was performed on a subset of patients (17) to evaluate pancreatic pathology (Table 3). Acute pancreatitis with PFC was observed in six (10.0%) whereas acute pancreatitis with necrosis in the tail in three (5.0%).

**Table 3:** Diagnostic imaging Values represented are frequency (percentage) for categorical data.

Diagnostic imaging	Descriptive statistic
Ultrasonography	
Bulky and edematous pancreas with peripancreatic fluid collection	6 (10.0%)
Bulky and inflamed pancreas with Peripancreatic fluid collection	2 (3.3%)
Bulky pancreas	14 (23.3%)
Edematous pancreas	11 (18.3%)
Edematous pancreas with necrosis in the tail	3 (5.0%)
Normal pancreas	19 (31.7%)
Pancreas not visualized	5 (8.3%)
Contrast enhanced computed tomography abdomen	
Acute pancreatitis	2 (3.3%)
Acute pancreatitis with bulky and edematous pancreas	1 (1.7%)
Acute pancreatitis with dilated pancreatic duct and peripancreatic fluid collection	1 (1.7%)
Acute pancreatitis with dilated pancreatic duct	1 (1.7%)
Acute pancreatitis with necrosis in the tail	3 (5.0%)
Acute pancreatitis with partial splenic vein thrombus	1 (1.7%)
Acute pancreatitis with peripancreatic fluid collection	6 (10.0%)
Acute pancreatitis with peripancreatic fluid collection with partial splenic vein thrombus	1 (1.7%)
Bulky and oedematous pancreas	1 (1.7%)
Not done	43 (71.7%)

**Figure 1:**
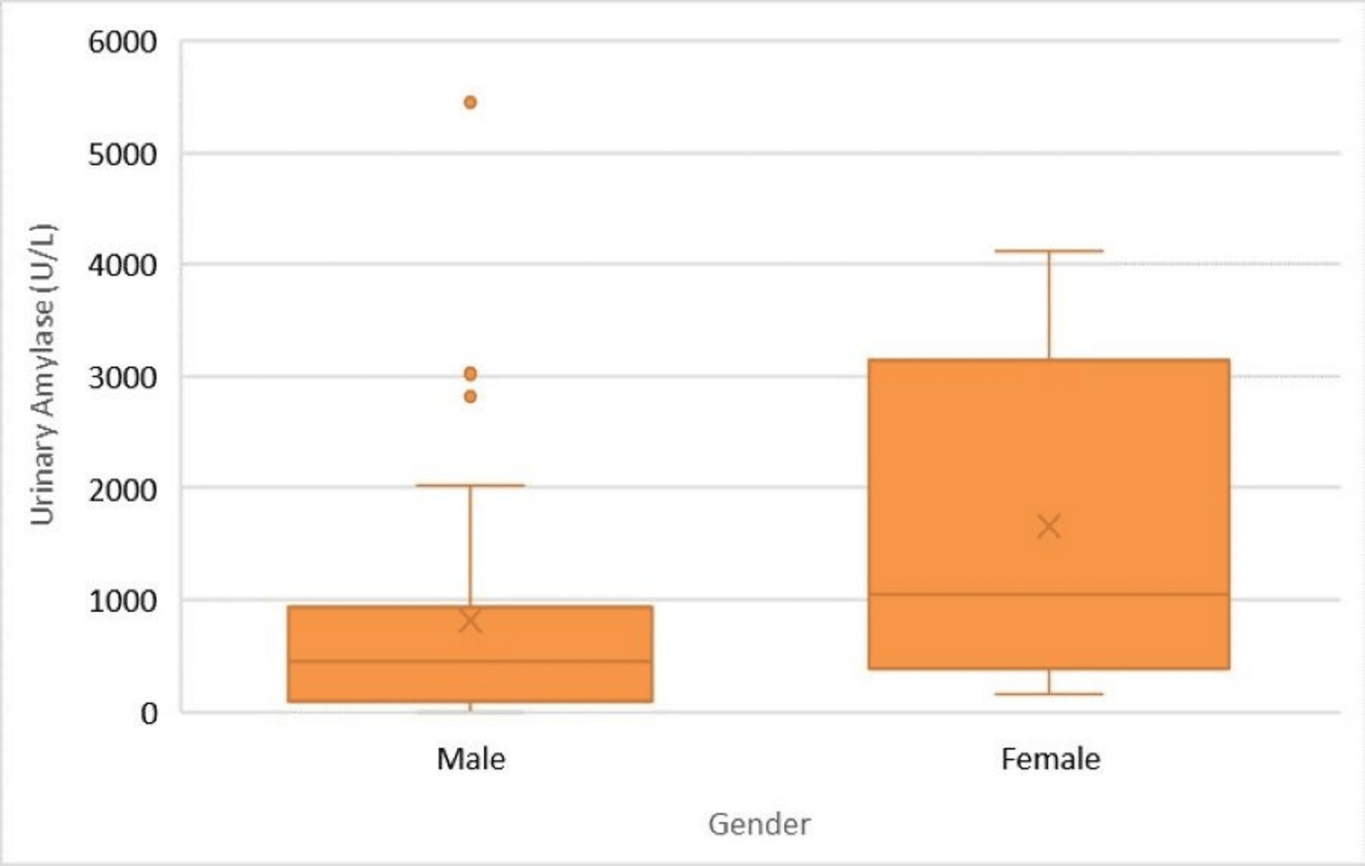
Boxplot of urinary amylase (U/L) by gender

The boxplot of urinary amylase levels (U/L) by gender provides a visual summary of the distribution and central tendencies of urinary amylase concentrations among male and female patients (Figure 1).

The median TLC for male patients was 9000 µL, with an IQR from 7,350 to 13,175 µL whereas female patients had a median TLC of 12,100 µL, with an IQR from 8,650 to 15,000 µL. The difference in TLC between male and female patients was statistically significant (p = 0.126), indicating no significant gender-based variation in TLC within this sample. The median total bilirubin level in male patients was 0.82 mg/dL (IQR: 0.51 - 1.34 mg/dL) whereas female patients had a median total bilirubin level of 0.79 mg/dL (IQR: 0.51 - 1.36 mg/dL), indicating no significant gender-based variation, suggesting comparable bilirubin metabolism or clearance in both male and female patients. Male patients exhibited a median serum amylase level of 249.5 U/L (IQR: 69.75 - 615 U/L) whereas the median serum amylase level in female patients was higher at 447.5 U/L (IQR: 297 - 1,549.5 U/L). The difference in serum amylase levels between genders was statistically insignificant (p = 0.071), although the higher median value in females suggests a trend toward higher enzyme activity.

The median serum lipase level among male patients was 477 U/L, with an IQR from 225.5 to 2,118.5 U/L whereas female patients had a median serum lipase level of 766.5 U/L (IQR: 199.5 - 2,696.5 U/L). No significant difference in serum lipase levels was observed between genders (p = 0.585), indicating similar lipase activity across the groups. Male patients showed a median urinary amylase level of 441 U/L, with an IQR of 96.25 to 927.75 U/L whereas the median urinary amylase level in female patients was significantly higher at 1,052 U/L (IQR: 377.5 - 3,153 U/L). The difference in urinary amylase levels between male and female patients was statistically significant (p = 0.039), suggesting a higher excretion rate of amylase in female patients (Table 4).

**Table 4:** Comparison of biochemical parameters by gender Values represented are median (interquartile range) for quantitative variables. Test used: Independent sample’s t-test (indicated by ‡) or Mann-Whiteney U test. p-value <0.05* statistically significant.

Gender	Male	Female	P-value
Total leukocyte count (µL)	9,000 (7,350 - 13,175)	12,100 (8,650 - 15,000)	‡0.126
Total bilirubin (mg/dL)	0.82 (0.51 - 1.34)	0.79 (0.51 - 1.36)	0.873
Serum amylase (U/L)	249.5 (69.75 - 615)	447.5 (297 - 1,549.5)	0.071
Serum lipase (U/L)	477 (225.5 - 2,118.5)	766.5 (199.5 - 2,696.5)	0.585
Urinary amylase (U/L)	441 (96.25 - 927.75)	1,052 (377.5 - 3,153)	0.039*

## Discussion

In India, a significant number of patients consume alcohol or have cholelithiasis due to which acute pancreatitis accounts for a significant portion of the admissions daily [5]. Acute pancreatitis can be caused due to various reasons, each is distinct from the others. On the other hand, cholelithiasis and alcohol consumption together are responsible for around 80% of the condition [5]. There are several markers and scoring systems that utilize said marker for stratification and planning of management of patients based on the severity of acute pancreatitis. However, most markers are non-specific and can be elevated in other acute inflammatory conditions. Serum amylase and lipase are used in conjunction in most hospitals in India. Yet, the journey to explore new, cost-effective, and reproducible markers continues. Urinary amylase is one such marker, easy to analyze, and more specific for acute pancreatitis.

The study cohort consisted of 60 patients with a mean age of 37.90 ± 10.55 years, predominantly male (83.3%), reflecting a significant gender disparity. This demographic distribution is consistent with previous research, which has shown a higher prevalence of acute pancreatitis in males, likely due to higher rates of alcohol consumption, a known risk factor for acute pancreatitis [5]. In contrast, a study from North India reported a female preponderance, with 68 out of 104 patients (65%) being female [6].

The high proportion of daily laborers (38.3%) among the study population may indicate a socioeconomic factor influencing the prevalence of acute pancreatitis. This finding aligns with studies suggesting that certain occupations with high alcohol use like daily laborers or physical strain are associated with an increased risk of Acute pancreatitis (AP) [7]. The clinical presentation was varied, with abdominal pain and vomiting being the most common complaints (45.0%). This is consistent with the literature, where abdominal pain and gastrointestinal symptoms are among the features of acute pancreatitis [8]. The low prevalence of co-morbid conditions such as DM (8.3%) and HTN (8.3%) in this cohort contrasts with studies that report a higher prevalence of chronic diseases among acute pancreatitis patients [9].

This discrepancy may be attributed to the relatively young age of the study population or geographic differences in disease patterns. The high prevalence of alcohol use (60.0%) and the presence of combined alcohol and smoking addiction (11.7%) in this cohort underscore the significant role of lifestyle factors in acute pancreatitis [7,10]. Previous studies have identified alcohol consumption as a major risk factor for acute pancreatitis, contributing to the pathogenesis of the disease through mechanisms such as pancreatic duct obstruction and direct pancreatic injury [7,10]. The correlation between smoking and acute pancreatitis has been less well-established, but combined substance use could exacerbate pancreatic damage [11].

The study's biochemical findings reveal variability in TLC, serum amylase, serum lipase, and urinary amylase levels. The median TLC of 9,400 cells/µL falls within the normal range, though it shows variability consistent with an inflammatory response. The median serum amylase level (311 U/L) and serum lipase level (498.5 U/L) indicate elevated pancreatic enzyme activity, corroborating the clinical presentation of AP. Elevated serum lipase is particularly indicative of acute pancreatitis, as it has greater specificity compared to amylase. The median urinary amylase level of 501 U/L further supports pancreatic involvement, with elevated levels suggesting ongoing enzyme secretion. This finding is consistent with studies that have demonstrated urinary amylase as a reliable marker for AP, especially when serum levels are normalizing [12,13]. Ultrasonography and CECT findings highlight the presence of various pancreatic conditions, with bulky pancreas and acute pancreatitis with PFC being notable. The prevalence of acute pancreatitis with PFC (10.0%) and acute pancreatitis with necrosis in the tail (5.0%) aligns with known complications of severe pancreatitis [14,15]. The analysis of gender-based differences revealed no significant variation in TLC, total bilirubin, serum lipase, and serum amylase levels.

However, urinary amylase levels were significantly higher in female patients (1,052 U/L) compared to male patients (441 U/L), suggesting a potential gender difference in enzyme excretion or metabolic rates. This finding warrants further investigation to understand the underlying mechanisms and implications for diagnosis and treatment.

Clinical implications

The findings of our study have significant implications for clinical practice. By validating the utility of urinary amylase as a diagnostic marker, our study suggests that integrating urinary amylase measurement could improve diagnostic accuracy and sensitivity for acute pancreatitis. This non-invasive approach may complement existing diagnostic methods and offer a valuable tool for early detection, particularly in cases where serum biomarkers are insufficient.

Limitations and future research

While our study presents promising results, it is essential to acknowledge its limitations. The first is an observational study design with a small sample size. Second, variability in enzyme measurements, lack of standardization in diagnostic criteria and timing, demographic differences, potential biases, and methodological inconsistencies are some of the limitations of studies on enzyme levels and their diagnostic accuracy. The variability in urinary amylase levels among different patients and the potential for contamination or interference with other urinary components must be considered. Future research should aim to standardize urinary amylase measurement techniques and explore its diagnostic efficacy in larger, diverse patient populations. Addressing these limitations in future research through standardized protocols, larger and more diverse study populations, and comprehensive diagnostic approaches can improve the reliability and applicability of findings in clinical practice.

## Conclusions

Urinary amylase proves to be a valuable diagnostic marker for acute pancreatitis, offering several advantages over traditional serum amylase measurements. Elevated urinary amylase levels, which rise within 24 hours of symptom onset and can remain elevated for several days, provide a sensitive indicator of acute pancreatitis, especially in cases with late presentation. This sensitivity is particularly beneficial when serum amylase levels are normal or when typical markers are inconclusive. In moderate to severe cases of acute pancreatitis, urinary amylase levels are significantly higher compared to mild cases, underscoring its utility in assessing the severity of the condition. Furthermore, urinary amylase is a useful diagnostic tool in atypical cases where serum amylase levels are normal or in conditions like macroamylasemia and hypertriglyceridemia, where serum amylase may be less relevant. Integrating urinary amylase measurement into clinical practice can enhance diagnostic accuracy and provide a more comprehensive approach to diagnosing acute pancreatitis. Its prolonged elevation and increased sensitivity make it a valuable adjunct, particularly in complex cases where serum biomarkers may fall short.

## References

[1] Igarashi H, Kawabe A, Ito T, Members for the Revision Committee of JPN Guidelines (2015). Drug therapy for acute pancreatitis. Suizo.

[2] Saxon E, Hinkley W, Vogel W (1957). Comparative value of serum and urinary amylase in the diagnosis of acute pancreatitis. AMA Arch Intern Med.

[3] Steinberg WM, Nauck MA, Zinman B (2014). LEADER 3--lipase and amylase activity in subjects with type 2 diabetes: baseline data from over 9000 subjects in the LEADER Trial. Pancreas.

[4] Whitcomb DC (2006). Clinical practice. Acute pancreatitis. N Engl J Med.

[5] Alkareemy EAR, Ahmed LA-W, El-Masry MA, Habib HA, Mustafa MH (2020). Etiology, clinical characteristics, and outcomes of acute pancreatitis in patients at Assiut University Hospital. Egyptian J Internal Med.

[6] Jha PK, Chandran R, Jaiswal P (2017). A clinical study of risk factors of acute pancreatitis in a tertiary care centre in north India. Int Surg J.

[7] Apte MV, Wilson JS (2003). Alcohol-related pancreatic damage: mechanisms and treatment. Alcohol Health Res World.

[8] Gapp J, Tariq A, Chandra S (2024). Acute Pancreatitis. https://www.ncbi.nlm.nih.gov/books/NBK482468/.

[9] Spagnolo DM, Greer PJ, Ohlsen CS (2022). Acute and chronic pancreatitis disease prevalence, classification, and comorbidities: a cohort study of the UK BioBank. Clin Transl Gastroenterol.

[10] Klochkov A, Kudaravalli P, Lim Y (2024). Alcoholic Pancreatitis. https://www.ncbi.nlm.nih.gov/books/NBK537191/.

[11] Ye X, Lu G, Huai J, Ding J (2015). Impact of smoking on the risk of pancreatitis: a systematic review and meta-analysis. PLoS One.

[12] Judal H, Ganatra V, Choudhary PR (2022). Urinary amylase levels in the diagnosis of acute pancreatitis: a prospective case control study. Int Surg J.

[13] Gupta J, Gupta A, Pao K (2017). Comparative study on sensitivity of serum and urinary amylase in the diagnosis of acute pancreatitis. J Med Sci Clin Res.

[14] Mohy-ud-din N, Morrissey S (2024). Pancreatitis. https://www.ncbi.nlm.nih.gov/books/NBK538337/.

[15] Zerem E (2014). Treatment of severe acute pancreatitis and its complications. World J Gastroenterol.

